# Improved Estimation of the Initial Number of Susceptible Individuals in the General Stochastic Epidemic Model Using Penalized Likelihood

**DOI:** 10.1155/2014/241687

**Published:** 2014-09-11

**Authors:** Changhyuck Oh

**Affiliations:** Department of Statistics, Yeungnam University, Gyeongsan, Gyeongbuk 712-749, Republic of Korea

## Abstract

The initial size of a completely susceptible population in a group of individuals plays a key role in drawing inferences for epidemic models. However, this can be difficult to obtain in practice because, in any population, there might be individuals who may not transmit the disease during the epidemic. This short note describes how to improve the maximum likelihood estimators of the infection rate and the initial number of susceptible individuals and provides their approximate Hessian matrix for the general stochastic epidemic model by using the concept of the penalized likelihood function. The simulations of major epidemics show significant improvements in performance in averages and coverage ratios for the suggested estimator of the initial number in comparison to existing methods. We applied the proposed method to the Abakaliki smallpox data.

## 1. Introduction

In any epidemic in a group of individuals, there is a subgroup of individuals who are not susceptible to a disease, that is, those immune to the disease naturally or by vaccination, as well as those not exposed to the disease owing to physical separation or other reasons. Therefore, estimation of the size of the initially susceptible population in the group might be pivotal; for example, see [1]. For the general stochastic epidemic model, [2–4] have dealt with estimating the initial number.

For the case where an epidemic is observed fully over a given time interval such that all infection and removal times are known, [2] provided martingale estimating equations to propose an estimator called the M-estimator of the initial number of susceptible individuals and its approximate variance. However, the M-estimator does not have the property of consistent coverage ratios for confidence intervals of the initial number of susceptible individuals. For the same conditions, [4] derived a likelihood function using the counting process theory after [5] to yield the maximum likelihood estimator called the k-MLE and its approximate variance. However, this likelihood function does not coincide with that given by [5] but with that obtained by using the survival function method of two earlier studies [6, 7]. The k-MLE better improves coverage ratios for confidence intervals than the M-estimator, but the problem of inconsistent coverage ratios remains. [3] extended the martingale procedure of [2] when only the removal times are observed. See [8] for a summary of the likelihoods of the completely observed data given parameters under the various model setups for the general stochastic epidemic model adopted by many researchers such as [5, 6, 9, 10].

Here, the first approach to improving the estimator of the initial number of susceptible individuals was to employ the likelihood function of [5]. A system of equations was derived from the log-likelihood function to find the MLEs of the infection rate and the initial number of susceptible individuals, and a normal limiting distribution was assumed to propose a corresponding approximate Hessian matrix. However, because simulations for the MLE give unstable results such as infinite values for the estimate of the initial number and low coverage ratios for confidence intervals such as the M-estimator, a method of penalized likelihood function is proposed. See [11] for an example of estimation using a penalized likelihood function.

Simulations were conducted to compare the proposed maximum penalized likelihood estimator called the p-MLE with the k-MLE and the M-estimator of the initial number of susceptible individuals. Then, the proposed method was applied to the Abakaliki smallpox data from Nigeria to compare results with the findings of [2, 4].

The rest of this paper is organized as follows: Section 2 presents the notations and the general stochastic epidemic model.  Section 3 describes the estimation methods.  Section 4 presents the simulation results.  Section 5 considers a numerical example, and Section 6 concludes with a discussion and concluding remarks.

## 2. Notations and the General Stochastic Epidemic Model

Notations very similar to those of [2, 4] were adopted for the spread of a susceptible-infected-removed infectious disease in a population whose individuals are mixing homogeneously. Suppose that the epidemic is observed over the time interval [0, *T*] in the population, whose size is *ν* + *a* at time *t* = 0, where *a* indicates infectious individuals and *ν* susceptible individuals. Let *S*(*t*) denote the number of susceptible individuals present at time *t*; *X*(*t*) the number of individuals infected up to and including time *t*, including the initial set of infectious individuals; *I*(*t*) the number of infectious individuals present at time *t*; *R*(*t*) the number of infected individuals removed up to and including time *t*; *β* the infection rate; *γ* the removal rate; and *G*_*t*_ the *σ*-algebra generated by the history {*S*(*u*), *I*(*u*); 0 ≤ *u* ≤ *t*}. The number of individuals who become infected and are removed by time *T*, respectively, is denoted by *n*_*I*_ ≤ *ν* and *n*_*R*_ ≤ *ν*. Note that when an individual becomes infected, the individual is assumed to be immediately infectious. Given *S*(*t*) and *I*(*t*), assume that the probability of a susceptible individual becoming infected and that of an infectious individual being removed within a small time interval (*t*, *t* + *h*] are given by *β*/*ν* *S*(*t*)*I*(*t*)*h* + *o*(*h*) and *γI*(*t*)*h* + *o*(*h*), respectively, such that the transition probability is

(1) Pr⁡⁡{X(t+h)−X(t)=1,  R(t+h)−R(t)=0 ∣ Gt} =βνS(t)I(t)h+o(h),Pr⁡⁡{X(t+h)−X(t)=0,  R(t+h)−R(t)=1 ∣ Gt} =γI(t)h+o(h),Pr⁡⁡{X(t+h)−X(t)=0,  R(t+h)−R(t)=0 ∣ Gt} =1−βνS(t)I(t)h−γI(t)+o(h).

The correction term *o*(*h*) becomes negligible when *h* is small; that is, *o*(*h*)/*h* → 0.

The process *S*(*t*), 0 ≤ *t* ≤ *T*, is assumed not to be observed, such that *ν* is not observable. However, the process *I*(*t*), 0 ≤ *t* ≤ *T*, is fully observable, such that the times at which individuals become infected are observable. The observation here includes the infection time for the infected individual and his or her removal time. Let **ϕ** = (*ϕ*_1_, *ϕ*_2_,…, *ϕ*_*n*_*I*__) be the ordered successive infection times observed over (0, *T*]. As indicated in [2], the number of individuals infected in [0, *T*] who are still susceptible at time *t* can be observed; that is, *S*_*T*_(*t*) = *X*(*T*) − *X*(*t*−). Note that although *I*(*t*) depends only on infection and removal times, *S*(*t*) depends on *ν*, as well as infection and removal times.

## 3. Derivation of the Estimators

First, consider the likelihood function of the parameters *β* and *ν* according to [5]

(2) L(β,ν ∣ ϕ)∝(∏j=1nIβνS(ϕj−)I(ϕj−)) ×exp⁡⁡{−βν∫0TS(t)I(t)dt},

where *S*(*ϕ*_*j*_^−^) and *I*(*ϕ*_*j*_^−^) denote the situation just prior to time *ϕ*_*j*_. The likelihood function (2) differs from that given by [4]

(3) $$\begin{array}{c} \left(\overset{n_{I}}{\underset{j=2}{\prod }}\frac{\beta }{\nu }S\left(\phi_{j}^{-}\right)I\left(\phi_{j}^{-}\right)\right)\exp\left\{-\frac{\beta }{\nu }\overset{T}{\underset{\phi_{0}}{\int }}S\left(t\right)I\left(t\right)dt\right\}, \end{array}$$

which is obtained under the same conditions as (2), except that the epidemic process is observed until all infectious individuals are removed, such that the likelihood function can be interpreted as obtained by observing the process over the interval (*ϕ*_1_, *∞*) for *β* and *ν*. Note that the likelihood functions (2) and (3) are both derived using the definition of a likelihood function in statistical physics based on the counting process theory.

Here, inferences are drawn for *ν* when the infection process *I*(*t*) is observed over a fixed time interval (0, *T*], which is a relaxation of the condition in [2, 4], where the epidemic is observed until it ceases.

The log-likelihood function is given by taking the logarithm of (2):

(4) L(β,ν ∣ ϕ)∝nI(log⁡⁡β−log⁡⁡ν)+∑j=1nIlog⁡⁡(S(ϕj)−1) −βν∫0TS(t)I(t)dt.

Here, we use the relation *S*(*ϕ*_*j*_^−^) = *S*(*ϕ*_*j*_) − 1. Note that the total number of susceptible individuals at time *u* is just *S*_*T*_(*u*) plus the number of susceptible individuals not infected by *T*:

(5) $$\begin{array}{c} S\left(u\right)=S_{T}\left(u\right)+\nu +a-X\left(T\right), \end{array}$$

where *S*_*T*_(*u*) = *X*(*T*) − *X*(*u* ^−^) denotes the number of individuals infected in (0, *T*] and still susceptible at time *u*. With (5) substituted into (4) to use information on those infected at the infection time, the following is obtained:

(6) L(β,ν ∣ ϕ)∝nI(log⁡⁡β−log⁡⁡ν) +∑j=1nIlog⁡⁡(ST(ϕj)+ν+a−X(T)−1) −βν{A1+(ν+a−X(T))A2},

where

(7) $$\begin{array}{c} A_{1}=\overset{T}{\underset{0}{\int }}S_{T}\left(t\right)I\left(t\right)dt,\;\;A_{2}=\overset{T}{\underset{0}{\int }}I\left(t\right)dt. \end{array}$$

Let the first partial derivatives of the log-likelihood function (4) with respect to *β* and *ν* be 0; thus, the system of two nonlinear equations is

(8) l1(β,ν)≡β−nIνg1(ν)=0,

(9) l2(ν)≡−nIA2g1(ν)+∑j=1nI1g2(ν,j)=0,

where

(10) $$\begin{array}{c} g_{1}\left(\nu \right)=A_{1}+A_{2}\left(a+\nu -X\left(T\right)\right),\\ g_{2}\left(\nu ,j\right)=S_{T}\left(\phi_{j}\right)+\nu +a-X\left(T\right)-1. \end{array}$$

Because there are no terms of *β* in (9), (9) can be solved with respect to *ν* to obtain the MLE $\hat{\nu }$ of *ν*. Then, the MLE $\hat{\nu }$ of *ν* can be plugged into (8) to get the maximum likelihood estimator of *β*:

(11) $$\begin{array}{c} \hat{\beta }=\frac{n_{I}\hat{\nu }}{g_{1}\left(\hat{\nu }\right)}, \end{array}$$

which is the same as the M-estimator of *β* in [2]. The two nonlinear equations are solved here separately to get solutions, whereas [4] maximizes his log-likelihood function of variables *ν* and *β*.

The Hessian matrix can be approximated by

(12) H(β^,ν^)=(−nIβ^2−A2ν^+g1(ν^)ν^2−A2ν^+g1(ν^)ν^22A2β^ν^2+nIν^2−2β^g1(ν^)ν^3−∑j=1nI1(g2(ν^,j))2),

assuming that the limiting distribution of $\hat{\beta }$ and $\hat{\nu }$ follows a normal distribution.

Equation (9) does not have a finite solution for some values of the observations of **ϕ**. To show this, we can first rewrite *l*_2_(*ν*) as

(13) $$\begin{array}{c} l_{2}\left(\nu \right)=\overset{n_{I}}{\underset{j=1}{\sum }}\frac{j-n_{I}+A_{1}/A_{2}}{{\left(\nu -j\right)}^{2}+\left(\nu -j\right)\left(j-n_{I}+A_{1}/A_{2}\right)}, \end{array}$$

by using the relationships *S*_*T*_(*ϕ*_*j*_) = *n*_*I*_ − *j* + 1 and *X*(*T*) = *n*_*I*_ + *a*. It is clear that when *A*_1_/*A*_2_ > *n*_*I*_ − 1, *l*_2_(*ν*) becomes positive such that the solution of (9) should be infinity. Here, we assumed *ν* > *n*_*I*_ without loss of generality.

When the number of the initial susceptible individuals is not large enough, the number of simulated epidemics for which the estimates of *ν* that did not exist cannot be ignored (Table 1). Therefore a method for improving the maximum likelihood estimator $\hat{\nu }$ is proposed by considering a penalized likelihood of (6):

(14) $$\begin{array}{c} L_{p}\left(\beta ,\nu \;|\;\phi \right)=L{\left(\beta ,\nu \;|\;\phi \right)}^{2}-\text{pen}\left(\nu \;|\;\phi \right), \end{array}$$

where

(15) $$\begin{array}{c} \text{pen}\left(\nu \;|\;\phi \right)=n_{I}A_{2}\left(\log g_{1}^{+}\left(\nu \right)-\log g_{1}\left(\nu \right)\right)+\log g_{2}\left(\nu ,1\right), \end{array}$$

for *g*_1_^+^(*ν*) = *A*_1_ + *A*_2_(*a* + *ν* − *X*(*T*) + 1), which modifies (9) to

(16) $$\begin{array}{c} l_{2}^{p}\left(\nu \right)\equiv -\frac{n_{I}A_{2}}{g_{1}^{+}\left(\nu \right)}+\overset{n_{I}}{\underset{j=2}{\sum }}\frac{1}{g_{2}\left(\nu ,j\right)}=0. \end{array}$$

Note that *g*_1_^+^(*ν*) is a modification of *g*_1_(*ν*) to make *l*_2_(*ν*) slightly bigger, and 1/*g*_2_(*v*, 1) is subtracted from *l*_2_(*ν*) to make it slightly smaller. The penalty function pen(*ν*∣**ϕ**) is heuristically chosen to penalize a large value of the estimate of *ν* for the log-likelihood (6). It can be shown that the denominator of the first derivative of pen(*ν*∣**ϕ**) with respect to *ν* is positive and that the numerator is given in the quadratic equation form *ν* ^2^ + *c*_1_ *ν* + *c*_2_ for constants *c*_1_ and *c*_2_ such that pen(*ν*∣**ϕ**) increases as *ν* increases with *ν* > *ν*_0_ for some finite value *ν*_0_ > 0. See [11] for a discussion in choosing a penalty function. Let the estimators of *ν* and *β* obtained by solving (8) and (16) be ${\hat{\nu }}_{P}$ and ${\hat{\beta }}_{P}$, respectively, and be called the p-MLE.

Note that the k-MLE ${\hat{\nu }}_{K}$ in [4] can be obtained by

(17) $$\begin{array}{c} l_{3}^{\phi }\left(\nu \right)\equiv -\frac{n_{I}A_{2}}{g_{1}^{\phi }\left(\nu \right)}+\overset{n_{I}}{\underset{j=1}{\sum }}\frac{1}{g_{2}\left(\nu ,j\right)}=0, \end{array}$$

instead of (16), where *g*_1_^*ϕ*^(*ν*) = *A*_1_^*ϕ*^ + *A*_2_(*a* + *ν* − *X*(*T*)) with *A*_1_^*ϕ*^ = ∫_*ϕ*_1__^*T*^ *S*_*T*_(*t*)*I*(*t*)*dt* to reduce the value of *l*_2_(*ν*) in (9).

The Hessian matrix can be approximated to

(18) H(β^P,ν^P)=(−nIβ^P2−A2ν^P+g1+(ν^P)ν^P2−A2ν^P+g1+(ν^P)ν^P22A2β^Pν^P2+nIν^P2−2β^Pg1+(ν^P)ν^P3−∑j=2nI1(g2(ν^P,j))2),

assuming that the limiting distribution of ${\hat{\beta }}_{P}$ and ${\hat{\nu }}_{P}$ follows a normal distribution. Therefore, the diagonal elements of ${\left(-H({\hat{\beta }}_{P},{\hat{\nu }}_{P})\right)}^{-1}$ can be used to give estimated standard errors $\hat{\text{se}}({\hat{\nu }}_{P})$ and $\hat{\text{se}}({\hat{\beta }}_{P})$, which may be used to construct approximate nominal 95% confidence intervals of *ν* and *β* as

(19) $$\begin{array}{c} {\hat{\nu }}_{P}\pm 1.96\hat{\text{se}}\left({\hat{\nu }}_{P}\right),\;\;{\hat{\beta }}_{P}\pm 1.96\hat{\text{se}}\left({\hat{\beta }}_{P}\right), \end{array}$$

respectively.

## 4. Simulations

A simulation study very similar to that of [2, 4] was conducted to compare the efficiency of the p-MLE relative to the k-MLE and the M-estimate. Here, populations of *ν* = 100, 250, 1000, and 5000 susceptible individuals and *a* = 5 initial infectious individuals are considered. In this simulation, *γ* = 1 and *β* = 1.5 and 1.3 were taken. Results were conditional on a major epidemic, and, therefore, following [2], only simulated epidemics with more than 20% of infected individuals were considered. For *β* = 1.3, epidemics with more than 40% of infected individuals were considered. The value of *T* was set to *∞* to compare simulation results with the findings of [2, 4]. For each combination of parameters, 1000 epidemics were simulated.

The number of simulated epidemics for which the maximum likelihood estimate $\hat{\nu }$ does not exist was counted (Table 1). The results resemble those of [2]. Furthermore, in each scenario, the following were computed: (i) $\text{av}({\hat{\beta }}_{P}),\;\;\text{av}\left({\hat{\nu }}_{P}\right),\;\;\mathrm{sd}({\hat{\beta }}_{P})$, and $\mathrm{sd}({\hat{\beta }}_{P})$ (averages and standard deviations of 1000 estimates of ${\hat{\beta }}_{P}$ and ${\hat{\nu }}_{P}$, resp.); (ii) $\text{av}(\hat{\text{se}}({\hat{\beta }}_{P}))$ and $\text{av}(\hat{\text{se}}({\hat{\nu }}_{P}))$ (averages of estimated approximate standard errors of 1000 estimates of ${\hat{\beta }}_{P}$ and ${\hat{\nu }}_{P}$, resp.); and (iii) *C*(*ν*) (percentage of 1000 nominal 95% confidence intervals containing *ν*), the coverage ratio, and av(*N*(*T*)) (average final size of simulated epidemics). The simulation results are presented in Table 2.

Among the 1000 simulations, there were no cases in which the estimate ${\hat{\nu }}_{P}$ was infinite; this held true for the k-MLE as well, as in [4]. In the comparison with the k-MLE and the M-estimator, there were substantial improvements in coverage ratios and averages of estimates of ${\hat{\nu }}_{P}$. The coverage ratios for the p-MLE were quite stable in comparison to those for the k-MLE and the M-estimator. An increase in the value of *ν* increased the coverage ratio such that all coverage ratios for *ν* = 5000 were close to the true confidence coefficient 0.95.

The average estimates of ${\hat{\nu }}_{P}$ were closer to the true value than those of ${\hat{\nu }}_{K}$, the k-MLE, for each combination of parameters. The standard deviations $\mathrm{sd}({\hat{\nu }}_{P})$ and the average of estimated approximate standard errors $\text{av}(\hat{\text{se}}({\hat{\nu }}_{P}))$ of 1000 estimates of ${\hat{\nu }}_{P}$ were quite close to each other in all cases. However, this was not the case for the k-MLE and the M-estimator.

In addition, the averages of 1000 estimates of ${\hat{\beta }}_{P}$ tended to increase to the true value with an increase in *ν*, and the standard deviations of estimates $\mathrm{sd}({\hat{\beta }}_{P})$ and the average of estimated approximate standard errors $\text{av}(\hat{\text{se}}({\hat{\beta }}_{P}))$ were also quite close to each other in all cases.

## 5. Application

The proposed method was applied to the Abakaliki smallpox data from Nigeria. [12] provided 29 infection times for infected individuals and the number of infectious individuals on each day of the epidemic in Abakaliki. The infectious period is assumed to be fixed at 7 days for every individual, and the latent period fixed at 13 days. The estimates ${\hat{\beta }}_{P}$ and ${\hat{\nu }}_{P}$, as well as corresponding standard errors, were obtained (Table 3). The function nleqslv in *R* was used to solve (16) for ${\hat{\nu }}_{P}$. The results indicated that both the estimate and estimated approximate standard error for ${\hat{\nu }}_{P}$ were less than those for ${\hat{\nu }}_{K}$ and ${\hat{\nu }}_{M}$. The estimate (33.88) and estimated approximate standard error for ${\hat{\nu }}_{P}$ (4.13) were close to those for ${\hat{\nu }}_{K}$ (35.27 and 6.70, resp.) but lower than those for ${\hat{\nu }}_{M}$ (42.12 and 37.15, resp.). The estimate of initial susceptible individuals was much lower than 120, the population size. Because of the assumption of homogeneous mixing between individuals, as in earlier studies [2, 4, 12, 13], this estimate was interpreted assuming that a number of individuals were not susceptible to the disease owing to natural immunity, vaccination, or isolation.

**Figure 1:**
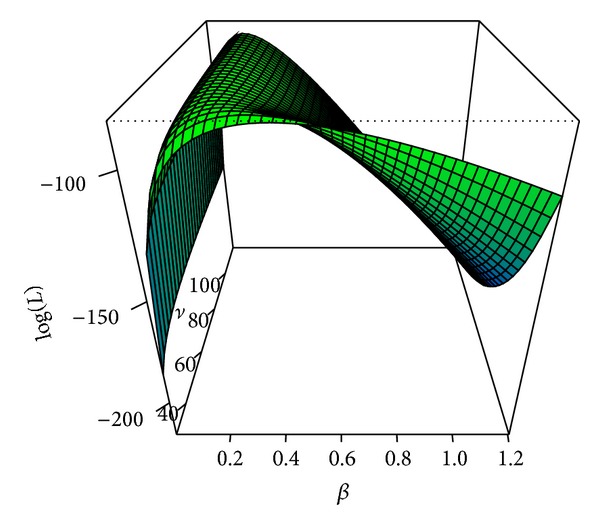
The log-likelihood function of *β* and *ν* for the Abakaliki smallpox data.

**Figure 2:**
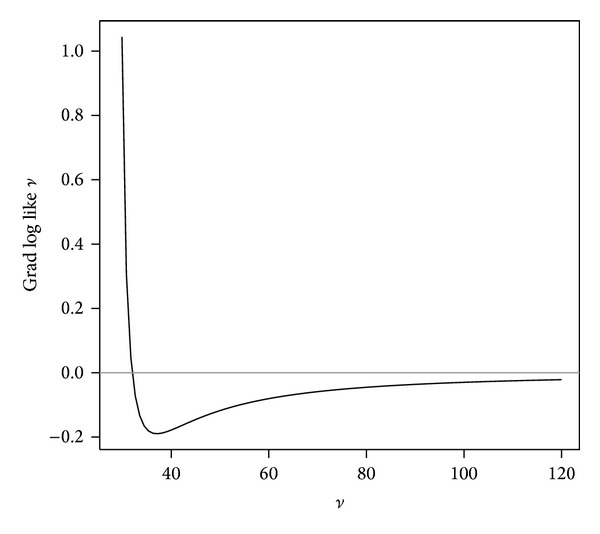
The gradient of the log-likelihood function of *ν* for the Abakaliki smallpox data.

Figure 1 shows the shape of the log-likelihood function of *β* and *ν*, which is similar to that in [4], and Figure 2 presents the gradient of the likelihood function of *ν* for the smallpox data.

## 6. Discussion and Conclusions

In any epidemic, the estimation of the initial number of susceptible individuals is of great interest. Although [5] derived a log-likelihood function for the initial number and the infection rate when the epidemic is fully observed over a given time interval, no study has investigated the properties of the MLEs. [4] considered the log-likelihood function to be obtained when the epidemic process is observed from the time of the first infection to the time when all infectious individuals are removed and derived the MLE of parameters of interest. [2] used a martingale framework to propose an estimator. The present study used the log-likelihood function of [5] and the relationship between *S* and *S*_*T*_ of  [2] to derive a system of equations for parameters and solved the system to obtain MLEs of parameters, instead of finding them as [4] did, by maximizing the log-likelihood function. An approximate Hessian matrix of estimators ${\hat{\nu }}_{P}$ and ${\hat{\beta }}_{P}$ was derived based on the assumption that the limiting distribution of ${\hat{\nu }}_{P}$ and ${\hat{\beta }}_{P}$ follows a normal distribution. The derivation of the limiting distribution of ${\hat{\beta }}_{p}$ and ${\hat{\nu }}_{p}$ can be a challenge.

Our modification of (9) can be considered the same as penalizing the likelihood function (4) with a suitable penalizing factor. Because there are various penalizing methods, another penalized likelihood function can be attempted for better estimators in a future study.

The simulations for the p-MLE provide a more stable result than the M-estimator and the k-MLE for unbiasedness, standard errors, and coverage ratios, and therefore, the proposed method can be used as a more reliable tool for estimating the initial number of susceptible individuals in a population.

## Figures and Tables

**Table 1:** The number of simulated epidemics for which the estimates of $\hat{\nu }$ did not exist for *a* = 5, *γ* = 1.0, replication = 1000, and endemic rate *ϵ*.

*ν*	(*β*, *ϵ*)		
	(1.5, 0.2)	(1.3, 0.2)	(1.3, 0.4)
100	41	85	28
250	3	26	6
1000	0	2	0
5000	0	0	0

**Table 2:** Simulation results for *a* = 5, *γ* = 1.0, replications = 1000, and *ϵ* = endemic rate.

(*β*, *ϵ*)	*ν*	av$\left({\hat{\beta }}_{P}\right)$	sd$\left({\hat{\beta }}_{P}\right)$	av$\left(\hat{\text{se}}({\hat{\beta }}_{P})\right)$	av$\left({\hat{\nu }}_{P}\right)$	sd$\left({\hat{\nu }}_{P}\right)$	av$\left(\hat{\text{se}}({\hat{\nu }}_{P})\right)$	*C*(*ν*)^a^	av(*N*(*T*))
(1.5,0.2)	100	1.34	0.20	0.25	98.7	30.6	36.9	89.0	57.6
	250	1.45	0.16	0.19	247.6	58.1	60.8	89.4	143.4
	1000	1.49	0.10	0.10	1004.1	117.9	118.2	93.1	578.6
	5000	1.50	0.05	0.05	5011.0	249.3	247.7	94.7	2907.1

(1.3,0.2)	100	1.21	0.18	0.24	93.4	34.0	43.7	86.5	48.4
	250	1.31	0.15	0.19	227.0	71.7	79.9	82.3	112.6
	1000	1.32	0.09	0.11	947.0	209.2	213.0	85.8	425.2
	5000	1.30	0.05	0.05	4965.1	540.7	536.1	92.5	2107.4

(1.3,0.4)	100	1.24	0.19	0.23	104.6	30.5	42.8	92.5	57.2
	250	1.32	0.15	0.18	251.8	63.6	76.1	90.7	131.4
	1000	1.33	0.09	0.10	1002.9	178.3	190.9	91.7	473.9
	5000	1.31	0.05	0.05	5020.8	488.4	492.0	94.6	2205.1

^a^Coverage ratio for *ν*.

**Table 3:** Estimates of the infection rate for initial susceptible individuals based on the Abakaliki smallpox data.

Parameter	Estimate	Standard error	Nominal 95% confidence interval
*β*	0.3133	0.0773	(0.1618, 0.4649)
*ν*	33.88	4.13	(25.79, 41.98)
